# Discovery and Characterization of a New Cold-Active Protease From an Extremophilic Bacterium via Comparative Genome Analysis and *in vitro* Expression

**DOI:** 10.3389/fmicb.2020.00881

**Published:** 2020-05-13

**Authors:** Amedea Perfumo, Georg Johannes Freiherr von Sass, Eva-Lena Nordmann, Nediljko Budisa, Dirk Wagner

**Affiliations:** ^1^GFZ German Research Centre for Geosciences, Helmholtz Centre Potsdam, Section Geomicrobiology, Potsdam, Germany; ^2^Polar Terrestrial Environmental System Division, Helmholtz Centre for Polar and Marine Research, Alfred Wegener Institute, Potsdam, Germany; ^3^Institute of Chemistry, Technische Universität Berlin, Berlin, Germany; ^4^Institute of Chemistry and Biology of the Marine Environment, Carl-von-Ossietzky Universität Oldenburg, Oldenburg, Germany; ^5^Institute of Chemistry, University of Manitoba, Winnipeg, MB, Canada; ^6^Institute of Geosciences, University of Potsdam, Potsdam, Germany

**Keywords:** bioprospecting, extremophilic bacteria, cold-active enzymes, genome mining, heterologous protein expression, microdiversity

## Abstract

Following a screening of Antarctic glacier forefield-bacteria for novel cold-active enzymes, a psychrophilic strain *Psychrobacter* sp. 94-6PB was selected for further characterization of enzymatic activities. The strain produced lipases and proteases in the temperature range of 4–18°C. The coding sequence of an extracellular serine-protease was then identified via comparative analysis across *Psychrobacter* sp. genomes, PCR-amplified in our strain 94-6PB and expressed in the heterologous host *E. coli*. The purified enzyme (80 kDa) resulted to be a cold-active alkaline protease, performing best at temperatures of 20–30°C and pH 7-9. It was stable in presence of common inhibitors [β-mercaptoethanol (β-ME), dithiothreitol (DTT), urea, phenylmethylsulfonyl fluoride (PMSF) and ethylenediaminetetraacetic acid (EDTA)] and compatible with detergents and surfactants (Tween 20, Tween 80, hydrogen peroxide and Triton X-100). Because of these properties, the P94-6PB protease may be suitable for use in a new generation of laundry products for cold washing. Furthermore, we assessed the microdiversity of this enzyme in *Psychrobacter* organisms from different cold habitats and found several gene clusters that correlated with specific ecological niches. We then discussed the role of habitat specialization in shaping the biodiversity of proteins and enzymes and anticipate far-reaching implications for the search of novel variants of biotechnological products.

## Introduction

Cold habitats are an extraordinary reservoir of biotechnological molecules such as enzymes (Cavicchioli et al., 2011), antimicrobials (Borchert et al., 2017), and biosurfactants (Perfumo et al., 2018) that can function under extreme conditions. In particular, cold-active enzymes, having catalytic activity below 25°C, can be used in energy saving bioprocesses. Catalysis at moderate temperature is an advantage also for the production of heat-sensitive bioproducts (e.g., drugs and therapeutics), the risk of contamination with mesophilic organisms is minimized as well as unwanted secondary products, and finally the bioprocess can be easily inactivated by raising the temperature (Cavicchioli et al., 2011; Santiago et al., 2016). Cold-adapted enzymes are also highly efficient because they can catalyze their reactions with chemical rates comparable to, and often exceeding, those of their warm-active counterparts (Struvay and Feller, 2012; Isaksen et al., 2016).

In this context, cold-active enzymes of microbial origin have tremendous biotechnological potential in a large range of markets such as detergents, food and beverages, textiles and can be used for specialty applications in molecular biology/research, pharmaceuticals, and diagnostics. Commercially available cold-active enzymes comprise mostly proteases and lipases, and to lesser extent amylases, cellulases, pectinases, mannanases, and others (Sarmiento et al., 2015). This market is expected to grow further in the coming years pushed forward by the rapid development of new technologies that both lead to the discovery of novel enzymes (e.g., genomics and metagenomics) and enable fine modifications of their chemical composition, structure and functional properties (e.g., genetic engineering and strain optimization or synthetic biology approaches) (Hoesl et al., 2011; Agostini et al., 2017).

The latest trend in the present omics-era is to carry out research on enzyme bioprospecting through environmental metagenomics (Berini et al., 2017) and functional genomics (Gerlt, 2017). Metagenome mining, especially if applied to microbial communities in extreme environments, holds great promise to discover entirely new classes of enzymes by-passing the technical challenges of culturing extremophilic microorganisms. However, the effective identification of promising enzymes from environmental metagenomes still suffers from low success rates, as highlighted by Ferrer et al. (2016). Therefore, a valid alternative is the functional genome mining approach. With the advances of high-throughput sequencing techniques, the number of bacterial genomes made publicly available has increased enormously, being at present in the order of tens of thousands (Ziemert et al., 2016). They obviously represent an extraordinary treasure trove of microbial biodiversity and functionality, where to search for genes coding for biomolecules and bioproducts of interest. In particular, comparative analysis of genome sequences is a relatively straightforward approach that allows the identification of target molecules across microorganisms, which differ for phylogeny, habitat or lifestyle. While this procedure, requiring known sequences of homologous enzymes, cannot lead to the discovery of entirely new molecules, it is still well suited to capture the molecular microdiversity that may arise from the adaptation to extreme environmental niches.

With this motivation, we have applied comparative genomics within the *Psychrobacter* genus to specifically identify and clone a gene encoding a cold-active protease in our isolate *Psychrobacter* strain 94-6PB, and further characterize the physicochemical properties of the expressed enzyme via *in vitro* analyses. *Psychrobacter* is a bacterium traditionally associated with low temperature environments (e.g., Siberian permafrost, Antarctic soil, seawater and sea-ice, deep-sea, glacial mud etc.) and is considered a model organism for studies on cold adaptation. Members of this genus have been shown to greatly differ in terms of both cold-adaptive traits and genome content (Zhang et al., 2017), and this can have important implications not only for the ecology of this organism but also for its use in industrial applications.

The biotechnological potential of *Psychrobacter* has been attracting increasing attention, for example for bioremediation treatments but also as a source of cold-active enzymes (e.g., lipases/esterases, proteases, β-lactamases, amylases, DNases) that can be used as catalysts in industrial bioprocesses (Dang et al., 2009). In this context, most of the research so far has specifically targeted lipases (most recently Novototskaya-Vlasova et al., 2012; Zhang et al., 2018), while in comparison little is known about proteases, the second industrially relevant enzyme. To contribute to closing the knowledge gap in this area, in this work we provide a description of a new cold-active protease from an Antarctic *Psychrobacter* together with a demonstration of an easy and effective experimental approach that can be also applied to the search and characterization of other types of microbial biomolecules.

## Materials and Methods

### Microorganisms and Cultivation Conditions

A collection of 33 bacterial strains previously isolated from a glacier forefield soil in the Larsemann Hills, East Antarctica (Bajerski and Wagner, 2013) was used in the present study (Supplementary Table S1). All microorganisms were routinely cultivated on R2A medium at a temperature of 10°C. *Psychrobacter* strain 94-6PB was selected for further characterization with regard to growth temperature range by measuring the optical density (OD_600__nm_) of cultures in marine broth medium (Difco) at 0, 4, 10, 15, 22, and 30°C. To obtain an accurate taxonomic identification, the 16S rRNA gene of bacterial strains was sequenced in almost full length with primers 27F and 1492R following standard protocols.

### On Plate Screening for Cold-Active Enzymes

Bacteria were screened for the synthesis of extracellular proteases and lipases at temperatures of 4, 10, and 18°C. Synthesis of proteases was assessed based on the original method by Frazier and Rupp (1928) by placing a volume of 10 μl of a pre-grown culture onto a calcium caseinate agar plate (Sigma-Aldrich, St. Louis, United States) and monitoring the formation of a clear halo around the colony during incubation for up to 4 weeks. As for lipases, bacterial strains were first screened for a general lipolytic activity by placing a volume of 10 μl of a pre-grown culture onto a R2A agar plate supplemented with sunflower oil (1% v/w) and rhodamine B (0.001% v/w) and monitoring under UV irradiation the formation of a fluorescent orange-pink halo around the colony (Beisson et al., 2000). Positive strains were then further screened for the synthesis of either esterases (EC 3.1.1.1) capable to degrade short chain fatty acids using tributyrin (C4) as substrate (1% v/w) or lipases (EC 3.1.1.3) capable to degrade long chain fatty acids using triolein (C18) as substrate (1% v/w).

### Comparative Genomic Analysis

Genome analysis and all further downstream analyses were performed on a selected bacterium, *Psychrobacter* strain 94-6PB. All genomes of *Psychrobacter* strains available at the NCBI database were individually searched for protease-coding genes (EC 3.4) and the retrieved sequences were screened with the program SignalP 4.1 (Petersen et al., 2011) for the presence of signal peptide cleavage sites to predict extracellularly released proteins. The resulting homologous sequences (Supplementary Table S2), including 500 bp-long untranslated 5′ and 3′ regions (UTR), were used for alignment with Clustal Omega and primers were designed with the program Primaclade (Gadberry et al., 2005) targeting conserved regions both within the coding sequence and in the UTRs. The complete list of the primers used for PCR amplification of the protease gene is reported in Supplementary Table S3.

### Gene PCR-Amplification and Sequencing

The target gene coding an extracellular protease was first amplified and sequenced in overlapping fragments, which were used to assemble the full sequence. Then, a specific pair of primers (P*prot*-F 5′-GCTTAACTAGTATCAACACTGCTG-3′ and P*prot*-R 5′-TCGTGCCGTACAGGTATAATCG-3′) was designed based on the obtained sequence and used to amplify the gene as single PCR amplicon. The PCR reaction mix consisted of 0.025U HotStart Plus Taq Polymerase, 0.2 mM dNTPs, 20 μM each primer, 3 mM MgCl_2_, 10 ng of DNA template, 2.5 μl of 10x PCR buffer and PCR grade water to final volume of 25 μl. Touch-Down PCR was performed as follows: initial denaturation at 95°C for 10 min, 11 cycles of denaturation at 95°C for 1 min, annealing starting at primer specific T_a_ (Supplementary Table S3) increased by 10°C for 1 min and then lowering by 1°C at each cycle, elongation at 72°C for 1 min, followed by 24 cycles of denaturation at 95°C for 1 min, annealing at primer specific T_a_ for 1 min, elongation at 72°C for 1 min, and final elongation at 72°C for 5 min. PCR products were checked for purity and molecular mass by gel electrophoresis.

Gene cloning was further applied in cases where it was not possible to obtain clear separation of the target band from unspecific products. Cloning of the gel-excised target band was performed using the TOPO TA Cloning Kit (Invitrogen, Carlsbad, CA, United States) with the cloning vector pCRTM2.1-TOPO vector Mach1^TM^-T1R according to the manufacturer’s instructions. Positive clones were picked from the plate and used for a screening PCR (initial denaturation at 96°C for 10 min, 35 cycles of denaturation at 95°C for 1 min, annealing at 55°C for 1 min, elongation at 72°C for 1.5 min, and final elongation at 72°C for 10 min). PCR products were purified by agarose gel band excision and sequenced using the M13 reverse primer. Sequencing was done at GATC Biotech (Köln, Germany).

### Gene Sequence Analysis and Protein Structure Prediction

The nucleotide sequence of the protease gene identified in our *Psychrobacter* strain 94-6PB (*prot94-6PB*) was compared with homolog sequences in other *Psychrobacter* organisms by multiple sequence alignment using Clustal Omega. The nucleotide sequence was translated into the corresponding amino acid sequence using the ExPASy online translation tool and the conserved domains were identified using NCBI’s conserved domain database (CDD). The 3-dimensional structure was then modeled using the SWISS-MODEL online tool and the PDB entry 6IQR with 35.74% sequence identity to P94-6PB_SP as template. For visualization, the PyMOL software (PyMOL Molecular Graphics System, Version 1.8 Schrödinger, LLC.) and CAVER 3.0 PyMOL plugin were used (DeLano, 2002; Chovancová et al., 2012).

### Heterologous Gene Expression and Purification of the Recombinant Protease

The protease-coding gene was PCR amplified both with (*prot94-6PB_SP*) and without (*prot94-6PB)* the signal peptide using the primer pairs prot-for1 (5′-GGTACATATGATGAAAAAACAAC CAGCAC-3′ with NdeI restriction site underlined) and prot-rev1 (5′-ATTTGTCTCGAGCAACTTGTCTTCTGGGCTAAC-3′ with XhoI restriction site underlined) and protSP-for1 (5′-GG CACACATATGGCAAACACTGATACTGAAGGC-3′ with NdeI restriction site underlined) and prot-rev1, respectively. The PCR reaction mix contained 0.5 μl (1 U) of Phusion DNA Polymerase (NEB), 0.25 μM dNTPs, 0.5 μM each primer, 25 ng of genomic DNA, 1.5 μl of DMSO, 10 μl of 5x HF buffer and PCR grade water to a final volume of 50 μl. PCR was performed with an initial denaturation at 98°C for 30 sec, followed by 30 cycles at 98°C for 10 sec, 60°C for 30 sec and 72°C for 1 min, and final elongation at 72°C for 10 min. Gel-purified PCR products as well as pET30b expression vector were digested at room temperature for 3 h with Fast Digest NdeI and XhoI restriction enzymes in FD green buffer (Thermo Fisher Scientific). Digested PCR products and expression vector pET30b were ligated using T4 DNA ligase and T4 DNA ligase buffer. Ligation was performed at 16°C for 18 h and the recombinant plasmids were transformed into chemically competent *E. coli* TOP10 cells (Thermo Fisher Scientific). Obtained cell colonies were screened for successful ligation by colony PCR using the primers T7 promoter-f (5′-TAATACGACTCACTATAGGG-3′) and T7 terminator-r (5′- GCTAGTTATTGCTCAGCGG-3′) and standard PCR conditions, and plasmids were sequenced to confirm the inserted protease target gene into pET30b expression vector.

Recombinant plasmids pET30b/*prot94-6PB* and pET30b/*prot94-6PB_SP* were transformed into *E. coli* BL21 (DE3) and *E. coli* ArcticExpress competent cells (Agilent Technologies). Overnight cultures of transformed cells were inoculated into ZYP5052 autoinduction medium (Studier, 2005) supplied with 1 mM kanamycin. Cells were grown at 37°C up to an optical density (OD_600__nm_) of 0.6 and afterward incubated at 13°C for 24 h. Cells were harvested (5000 × *g* at 4°C for 20 min), resuspended in 15 ml of wash buffer (50 mM Na_2_HPO_4_/NaH_2_PO_4_, 300 mM NaCl, pH 7.5) and incubated with lysozyme (300 μg/ml), DNaseI and RNase (10 μg/ml each) for 1 h on ice. Cells were disrupted using an M-110L Microfluidizer processor (Microfluidics, Westwood, MA, United States), yielding the crude extract. Cell debris was then removed by centrifugation (15,000 × *g* at 4°C for 60 min) and the supernatant was filtered (0.45 μm) to recover the cell lysate. P94-6PB protein purification was performed on a ÄKTA pure system (GE Healthcare, Munich, Germany) using Ni-NTA columns (GE Healthcare). The hexahistidin-tagged proteins were eluted with an imidazole gradient (0–500 mM) and protein-containing fractions were dialyzed against the storage buffer (15 mM Na_2_HPO_4_/NaH_2_PO_4_, 137 mM NaCl, pH 7.5). During cell disruption and purification, protein samples were analyzed by SDS polyacrylamide gel electrophoresis (SDS-PAGE) with Coomassie blue staining. Finally, protein concentration was determined using the Bradford assay and bovine serum albumin (BSA) as standard. Correct protein mass was verified by electrospray ionization mass spectrometry (LC-ESI-TOF-MS, Agilent 1260 HPLC coupled with Agilent 6530 Accurate-Mass Q-TOF mass spectrometer; Agilent, Santa Clara, CA, United States).

### Protease Activity Assay

Activity assay was performed in a 100 μl volume using azocasein (Sigma-Aldrich) as substrate following the method described by Tomarelli et al. (1949). Briefly, hydrolysis of the casein releases the azo dye into the medium where it is detected by absorbance at 440 nm. Therefore, azocasein (final concentration 10 mg/ml) was preheated/cooled to the assay temperature and the reaction was started with the addition of the P94-6PB solution (final concentration 1 mg/ml). The mixture was shaken for 30 min and stopped with 100 μl Trichloroacetic acid (TCA) [10 % (w/v)]. In order to ensure a quantitative separation of precipitate and supernatant, the solution was centrifuged at 16,000 × g, for 60 min at 4°C, and 100 μl of the supernatant was mixed with 100 μl 2 M NaOH. The activity was determined by absorption measurements at 440 nm. For each condition, a blank measurement was performed. Here, 100 μl TCA [10% (w/v)] were added prior enzyme addition. All measurements were performed in triplicates.

### Temperature- and pH-Range Activity Profile

For pH-dependent measurements, the protease activity assay was performed at 37°C. Reaction buffer was either Tris-buffer (0.2 M Tris, 0.1 M acetic acid, 0.1 M 2-N-Morpholino Ethane sulfonic acid, 0.01 M CaCl_2_, pH 5–8) or Glycine buffer (0.2 M Glycine, 0.01 M CaCl_2_, pH 9-11). For temperature-dependent measurements, the protease activity assay was performed at pH 9.0 using a Tris-buffer (0.1 M Tris, 0.1 M Glycin, 0.1 M NaCl, 0.01 M CaCl_2_) at different temperatures ranging from 5 to 50°C.

### Irreversible Thermal Inactivation

Enzyme solution (2 mg/mL) was incubated at different temperatures ranging from 5 to 50°C for 1 h and subsequently stored on ice for 30 min. Precipitated protein was spinned down and the supernatant was used for the protease activity assay as described above at 30°C and pH 9.0.

### Effect of Enzyme Inhibitors

The effect of enzyme inhibitors was investigated at 30°C and pH 9.0 using a Tris-reaction buffer (0.1 M Tris, 0.1 M Glycine, 0.1 M NaCl, and 0.01 M CaCl_2_). Urea, Dithiothreitol (DTT), β-mercaptoethanol (β-ME) and Ethylenediaminetetraacetic acid (EDTA) were dissolved in the reaction buffer, Phenylmethylsulfonyl fluoride (PMSF) was dissolved in methanol (0.5 M each). Protease solution was incubated with 2 or 5 mM of the respective inhibitor for 30 min on ice to minimize any temperature-related structural instability. As a control, the protease was incubated with the same volume of buffer/methanol. Reaction was started with the addition of the pre-incubated enzyme solution to the azocasein solution and the assay was performed as described above.

### Effect of Surfactants and Detergents

The effect of surfactants and detergents was investigated at 30°C and pH 9.0 using a Tris reaction buffer (0.1 M Tris, 0.1 M Glycine, 0.1 M NaCl, and 0.01 M CaCl_2_). Tween 20 (Sigma-Aldrich), Tween 80 (Sigma Aldrich), sodium dodecylsulfate (SDS; Roth), Triton X-100 (Roth) and the oxidizing agent H_2_O_2_ (Roth) were prepared as 20% stock solutions in the reaction buffer. Protease solution was incubated with 1 or 5% of the respective chemical for 30 min on ice. As a control, the protease was incubated with the same volume of buffer. Reaction was started with the addition of the pre-incubated enzyme solution to the azocasein solution and the assay was performed as described above.

### Phylogenetic and Pairwise Identity Analysis

Percentage pairwise identity was computed with EBI’s MUSCLE tool and visualized as Heat-Map with Morpheus software^1^. Evolutionary relationships were determined by using the Maximum Likelihood method based on the Tamura-Nei model. Initial tree(s) for the heuristic search were obtained automatically by applying Neighbor-Join and BioNJ algorithms to a matrix of pairwise distances estimated using the Maximum Composite Likelihood (MCL) approach, and then selecting the topology with superior log likelihood value. All positions containing gaps and missing data were eliminated. Evolutionary analyses were conducted in MEGA7 (Kumar et al., 2016).

### Sequence Accession Number

The nucleotide sequence of the gene *prot94-6PB* has been submitted to GenBank database under the accession number MN606318.

## Results

### Abundance and Biodiversity of Bacteria With Enzymatic Activity in Glacier Forefields

**FIGURE 1:**
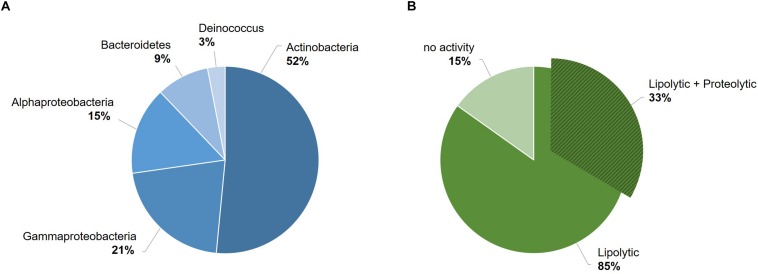
Overview of the bacterial strain collection from the Larsemann Hills (East Antarctica) used in this study for the search of cold-active enzymes. Bacterial isolates were assessed for both biodiversity (i.e., taxonomic affiliation) **(A)** and extracellular enzymatic activities at low temperatures (4–18°C) **(B)**.

The 33 bacterial isolates from glacier forefield soils in the Larsemann Hills (East Antarctica) used in this study were largely represented by Actinobacteria (17/33; 52%), particularly of the *Arthrobacter* genus, followed by Gamma-proteobacteria (7/33; 21%), Alpha-proteobacteria (5/33; 15%), Bacteroidetes (3/33; 9%) and Deinococcus (1/33; 3%) (Figure 1A). Among these isolates, 85% (28/33) screened positive for enzymatic activity, while 15% (5/33) showed no activity. All active strains were found to produce lipases and 33% (11/33) produced both lipases and proteases. The latter ones comprised mostly strains of *Arthrobacter*, *Pseudomonas* and *Psychrobacter* strain 94-6PB (Figure 1B). Among the strains with lipolytic activity, 46% (13/28) produced only lipases (degradation of C18 fatty acids), 14% (4/28) only esterases (degradation of C4 fatty acids), 21% (6/28) produced both and for 18% (5/28) it was not possible to discriminate (Supplementary Table S1 and Supplementary Figure S1).

### *In silico* Analysis and Gene Identification of P94-6PB Protease

Representing a model organism for cold-adaptation, *Psychrobacter* strain 94-6PB was selected for further characterization. Based on the almost full-length 16S rRNA gene sequence, this strain was found closest to *Psychrobacter glacincola* (99%). It was capable to grow in a temperature range from 0 to 20°C (subzero temperatures were not tested) and showed highest growth rates at 15°C (μ_max_ 0.07 h^–1^). Little growth was observed at 30°C.

**FIGURE 2:**
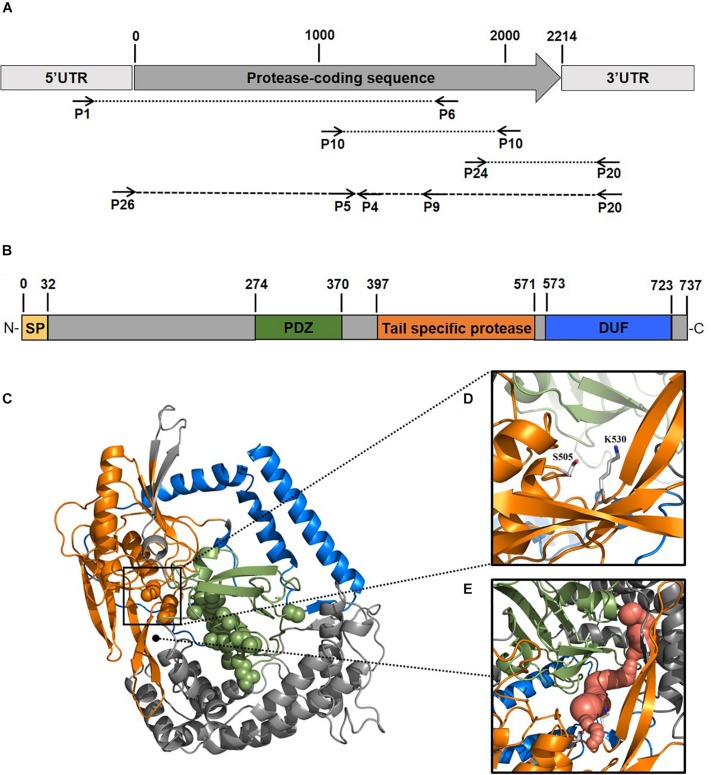
Scheme of the experimental approach used to identify a new protease from the Antarctic bacterium *Psychrobacter* sp. 94-6PB. Target protease-coding gene was PCR-amplified in our strain using a combination of primers designed based on comparative genome analysis across *Psychrobacter* organisms **(A)**. Sequence-based prediction of the domains of the target enzyme (SP, signal peptide; PDZ domain for substrate recognition; tail-specific protease domain with catalytic function; DUF, domain of unknown function) **(B)**. Predicted 3D-macromolecular structure of the target protease **(C)**, with catalytic dyad at position S505 and K530 **(D)** and a connecting tunnel to the PDZ domain for substrate recognition **(E)**.

To better elucidate the biotechnological potential of its proteolytic enzymes, we set out to identify the gene sequence of an extracellular protease, clone it in heterologous hosts and characterize the physicochemical properties and activities of the purified enzyme *in vitro*. Because strain 94-6PB had no sequenced genome, we PCR-amplified and sequenced the protease-target gene using primers designed on the basis of comparative analysis of homologous genes in other *Psychrobacter* organisms. Search on the NCBI database for all available *Psychrobacter* genomes, both complete and as scaffolds/contigs, revealed that homologous sequences of a gene coding for an extracellular peptidase (i.e., signal site for extracellular translocation predicted to be at the amino acid in position 31/32) were present in 12 genomes (Supplementary Table S2). Multiple sequence alignment of both gene and protein sequences showed that most of the peptidases shared similarity >80%. Highly conserved regions were identified within the peptidase-coding gene of all strains as well as in the flanking untranslated regions (5′-UTR and 3′-UTR) and they were used as target for primer design and PCR amplification. Initially, the gene was amplified in 3 overlapping fragments using the following primer combinations: P1-F/P6-R (product size 2080 bp), P10-F/P10-R (product size 1156 bp) and P24-F/P20-R (product size 555 bp). In addition, internal primers P5-F, P4-R and P9-R were used for sequencing. Finally, using primers P26-F and P20-R, a 2471 bp-long amplicon was obtained without any secondary products. Subsequent sequencing confirmed the full peptidase-coding gene including parts of the 5′ UTR (54 bp upstream) and 3′ UTR (203 bp downstream) (Figure 2A, Supplementary Table S3 and Supplementary Figure S2).

### Protease Sequence Analysis and Prediction

The gene coding sequence resulted to be 2214 bp long and its translated polypeptide was predicted, by the Expasy translate tool, to be 737 aa long, with an estimated molecular size of approximately 80 kDa (Figure 2B). Analysis with the NCBI’s CDD tool confirmed the identification of a serine peptidase belonging to the S41 superfamily (E.C. 3.4.21). The protein was predicted to consist of: (i) transmembrane region, (ii) PDZ domain containing a protein-binding site involved in substrate recognition; (iii) tail-specific protease domain with catalytic activity; and (iv) a conserved domain of unknown function (DUF) at the C-terminal region of the protease (Figure 2B). Modeling of the 3-dimensional structure (Figure 2C) resulted in a globular protein consisting of 18 α-helices and 19 β-strands. The substrate-binding site showed to be well exposed on the surface, while the catalytic dyad was found to be in the inner part of the enzyme. Superimposition of the modeled structure of P94-6PB_SP and the template 6IQR showed the catalytic dyad at position S505 and K530 (Figure 2D). Besides, a tunnel connecting the PDZ domain for substrate recognition and the catalytic dyad could be also detected (Figure 2E).

### *In vitro* Expression and Purification

**FIGURE 3:**
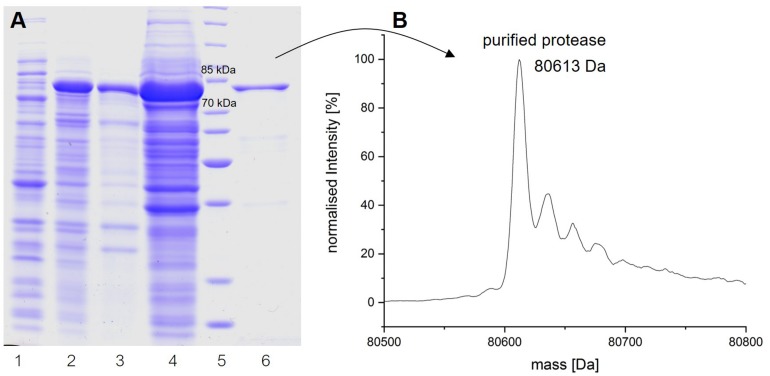
Analysis of the expression and purification of P94-6PB protease. Analysis on 12% SDS-PAGE of enzyme induction, release and purification **(A)**: non-induced (1), induced (2), lysate (3), crude extract (4), protein ladder (5) and purified enzyme (6). Electrospray ionization mass spectrometry (LC-ESI-TOF-MS) analysis and deconvoluted mass spectrum of the purified eluate **(B)**.

To obtain insights into the physicochemical properties and activity of the 94-6PB protease, we first worked to express the enzyme in heterologous hosts. The PCR product containing the protease sequence (with and without signal peptide) was cloned into pET30b expression vector and the recombinant plasmid was transformed into both *E. coli* BL21 (DE3) and *E. coli* ArcticExpress (DE3). The latter strain, co-expressing cold-active chaperonins, Cpn10 and Cpn60 from *Oleispira antarctica*, that assist in the folding of proteins at low temperatures, was expected to be particularly suited for molecules from psychrophilic organisms. However, expression tests using the two strains, different kanamycin concentrations as well as different media and cell disruption methods did not exhibit higher expression levels of the protease using the *E. coli* ArcticExpress strain. Highest protein yields (33 mg/l cultivation) could be obtained by cultivating BL21 (DE3) in ZYP medium supplemented with 50 μg/ml kanamycin. Finally, the highest protein yields after his-tag purification, 1.65 mg/50 ml cultivation, were obtained using *E. coli* BL21(DE3) as expression strain with no addition of kanamycin and combined with the microfluidizer as extraction method. The his-tagged protease could be successfully expressed and purified via affinity chromatography (Figure 3A). To verify the purified protease, we performed mass analysis and detected a peak corresponding to mass 80613 Da for the protease without signal peptide, which is matching with the calculated mass (Figure 3B). A summary of the purification steps of the protease is reported in Table 1.

**TABLE 1:** Purification of the extracellular protease produced by *Psychrobacter* sp. P94-6PB.

Purification step	Fraction volume (ml)	Protein concentration (mg/ml)	Inserted protein amount in activity assay (mg)	Total activity (U)	Specific activity (U/mg)	Purification fold	Yield (%)
Crude extract	80	70	3.5	191	54.6	1	100
Ni-NTA	37.5	2	0.1	56	560.0	10	29

*One unit of activity was defined as an increase of 0.01 in absorbance units at 440 nm in the azocasein assay.*

### Temperature- and pH-Range Activity and Stability

**FIGURE 4:**
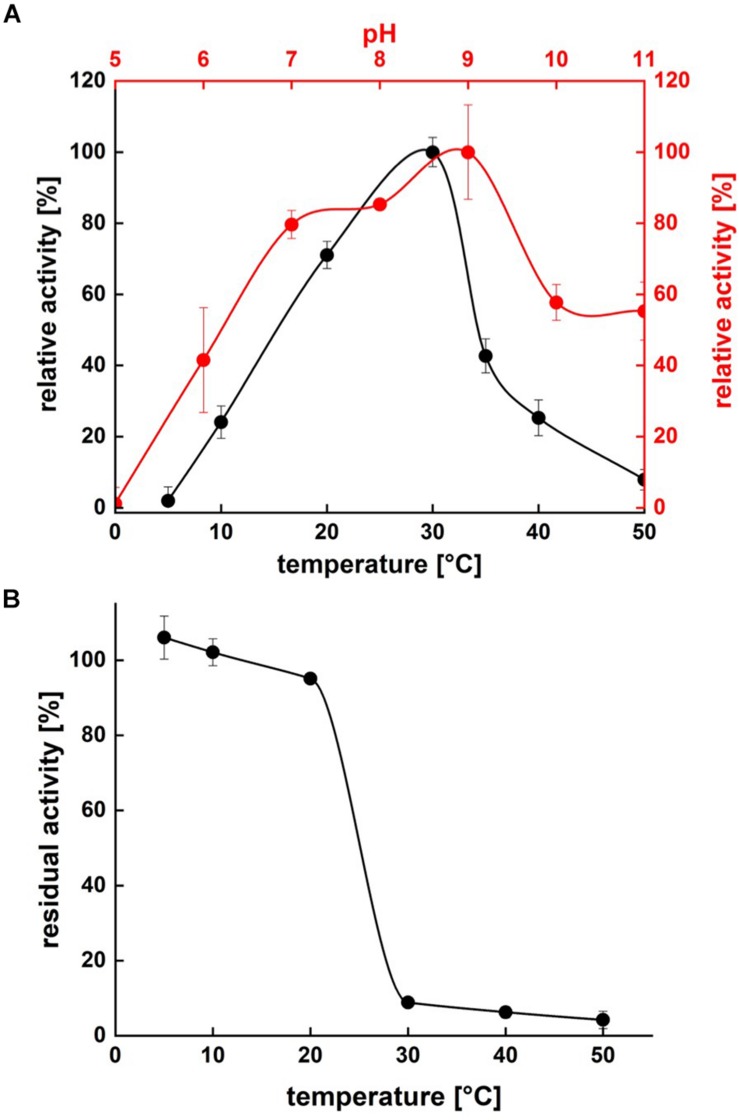
Effect of temperature and pH on P94-6PB protease activity **(A)** and stability **(B)**. Overview of temperature and pH range of the activity and stability of P94-6PB protease. Relative activity was quantified using the azocasein assay.

Protease activity assays using azocasein as substrate demonstrated highest activity of P94-6PB at pH 9.0. At higher pH values (pH 10.0 and 11.0), relative protease activity was still above 50%, whereas under acidic conditions (pH 5.0), P94-6PB exhibited no protease activity (Figure 4A). Thus, investigation of temperature-dependent protease activity was performed at pH 9.0. Here, P94-6PB exhibited highest activity at 30°C. At 20°C, we still observed a relative protease activity of 71%. Reducing the temperature to 10°C yielded a relative activity of 24%. At 5 and 50°C, the protease exhibited relative protease activities less than 10% (Figure 4A). With regard to the effect of temperature to the enzyme stability, P94-6PB was stable at temperatures ≤20°C. When incubated at higher temperatures, large precipitation of the protease was observed, and the residual activity dropped to less than 10% (Figure 4B). Overall, our data indicated that P94-6PB is an alkaline cold-active protease.

### Effect of Inhibitors and Detergents on Enzyme Activity

**FIGURE 5:**
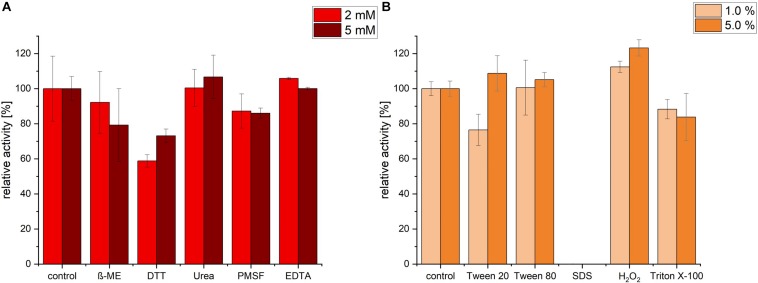
Effect of inhibitors, detergents and surfactants on P94-6PB protease activity. Relative activity measured via the azocasein assay after pre-incubation (30 min) with inhibitors (β-mercaptoethanol (β-ME), Dithiothreitol (DTT), Urea, Phenylmethylsulfonyl fluoride (PMSF) and Ethylenediaminetetraacetic acid (EDTA) at 2 and 5 mM concentration) **(A)** and detergents and surfactants (Tween 20, Tween 80, sodium dodecylsulfate (SDS), Hydrogen peroxide (H_2_O_2_) and Triton X-100, at 1.0 and 5.0% v/v concentration) **(B)**.

Protease activity was measured after pre-incubation with a range of different inhibitors, detergents and surfactants. Incubation with EDTA and Urea had no significant change in protease activity. In contrast, ß-mercaptoethanol (β-ME), DTT and PMSF exhibited an inhibiting effect on the protease. The highest decrease in activity was detected in presence of 2 mM of DTT (relative activity of 58.78%), however, increasing DTT concentration had no further decrease effect, possibly because maximal inhibition was already achieved with 2 mM DTT (Figure 5A). We noticed that, under the conditions (time, temperature and pH) we used, PMSF exerted only a partial inhibition of P94-6PB.

Incubation with the detergent Tween 80 had no significant impact on the protease activity, whereas Triton X-100 treatment induced a slight decrease of the relative activity. Incubation with 1% Tween 20 caused a reduction of activity by approximately 24%; however, increasing Tween 20 concentration to 5%, had a slightly positive effect. Apparently, P94-6PB activity was highly dependent on the detergent concentration, as similarly observed by others (Abdel-Hamed et al., 2016). Incubation with 1% SDS caused the complete loss of protease activity. Finally, we could observe that pre-incubation with 1 and 5% of the oxidizing agent H_2_O_2_ resulted in an increase of the relative activity by 12 and 23%, respectively (Figure 5B). Here, it is likely that H_2_O_2_ contributed to make azocasein more susceptible to proteolytic degradation (Fligiel et al., 1984).

### Enzyme Microdiversity in *Psychrobacter* Organisms From Different Cold-Habitats

**FIGURE 6:**
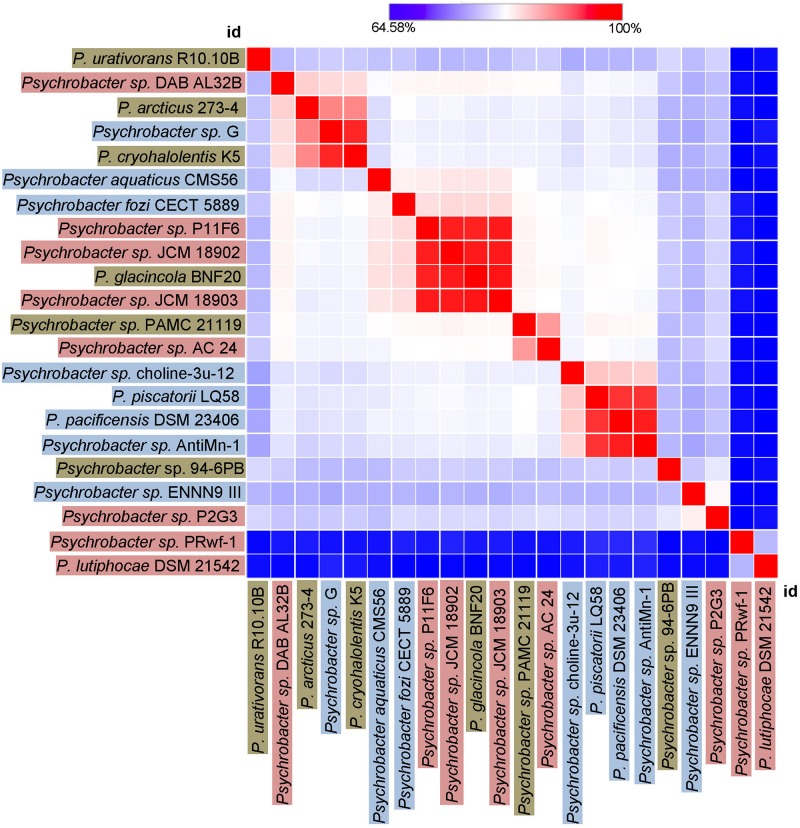
Heat-Map showing percentage identity of the studied protease across the *Psychrobacter* genus. Homologous gene sequences coding for the target protease in 22 strains of *Psychrobacter* sp. (including our strain 94-6PB) are used as entry points of the Heat-Map. Each strain identifier (id) is reported as row and column, and color-coded according to the ecological source (terrestrial-brown, aquatic-blue and host associated-pink). A color bar indicates the correspondence between pairwise identities and the colors displayed in the matrix.

Finally, we sought to investigate how genetically different is the protease of our strain 94-6PB compared with homologous sequences of other 21 *Psychrobacter* strains from different ecological niches subdivided in three main groups: terrestrial habitats (e.g., soil and permafrost, Arctic and Antarctic), aquatic habitats (e.g., marine water, polluted and pristine, cold and temperate) and host-associated (e.g., skin and intestinal, animals, fish, tunicates). The phylogenetic reconstruction, inferred by using the Maximum Likelihood method based on the Tamura-Nei model, showed a general tendency of the enzyme sequences to cluster on the basis of strain-specific habitat, and this was also reflected in the percentage identity matrix (Figure 6 and Supplementary Figure S3). Strains of aquatic origin such as *Psychrobacter* sp. choline-3u-12, *P. piscatorii* LQ58, *P. pacificensis* DSM 23406 and *Psychrobacter* sp. AntiMin-1 clustered together, and particularly the three latter ones, all isolated from deep seawater in the Pacific ocean, shared high sequence similarity (97–98%). Similarly, also terrestrial strains showed a high degree of relatedness, particularly *P. arcticus* 273-4 and *P. cryohalolentis* K5 (91%), both from permafrost of the Kolyma region in Siberia. However, also *Psychrobacter* sp. G, a seawater isolate, showed high similarity (91 and 98%, respectively) with both permafrost strains. Host-associated *Psychrobacter* sp. JCM 18902, *Psychrobacter* sp. JCM 18903 and *Psychrobacter* sp. P11F6, all isolated from cold/frozen marine organisms shared also high sequence similarity (98%). The overall similarity among all sequences of the serine peptidase enzyme was approximately 80 ± 5%, with some strains such as *Psychrobacter* sp. PRwf-1 and *P. lutiphocae* DSM 21542, both host-associated, being rather dissimilar from the others and having sequence similarity as low as 66 ± 3%. The enzyme of our isolate *Psychrobacter* sp. 94-6PB (from Antarctic soil) was closest (80% sequence identity) to *P. urativorans* R310.10 (from Antarctic soil) and *Psychrobacter* sp. P2G3 (host-associated in the Arctic) (Figure 6). While more data also on other enzyme families need to be considered, the role of environmental conditions in shaping the molecular diversity, hence the functionality, may have important implications for the search and/or selection of novel biocatalysts from natural sources.

## Discussion

Fast-growing industrial demand for sustainable bioproducts with unique functionalities (e.g., performing under harsh conditions) drives research in the field of bioprospecting toward the continuous search for novel organisms and biomolecules, and also for new methodologies enabling their discovery. Here, we report on the characterization of a cold-active alkaline protease from an Antarctic bacterium, *Psychrobacter* sp. 94-6PB. This enzyme is active over a wide range of temperatures (10–50°C) and pH values (6–11) but performs best at 20–30°C and pH 7-9. It is largely unaffected by the major protease inhibitors and is compatible with most of common detergents and surfactants; only SDS exerted a substantial negative effect. However, the partial tolerance of P94-6PB against the well-known serine protease inhibitor PMSF needs further investigation.

Collectively, these physicochemical properties make it highly suitable for applications especially as detergent additive. Today, companies worldwide are strongly committed to policies for sustainable laundry, which include lowering the washing temperature while maintaining washing performance unaltered. As an example, in 2014 DuPont and P&G won the Sustainable Bio Award following a collaboration to develop energy-saving laundry detergents. Despite the fact that new enzymes of this type are highly attractive for applications (Al-Ghanayem and Joseph, 2020), only 7% of the studies so far reported on proteases produced by psychrophilic bacteria, while the large majority (62%) still focused on proteases from mesophiles (Salwan and Sharma, 2019). Known cold-active proteases of bacterial origin, e.g., *Bacillus subtilis* WLCP1, *Pseudoalteromonas arctica* PAMC 21717, *Chryseobacterium* sp., and *Stenotrophomonas maltophilia* MTCC 7528 all share similar properties with the 94-6PB protease from *Psychrobacter* sp., having optimum pH at 9–10 and optimum temperature at 10–30°C (reviewed by Joshi and Satyanarayana, 2013; Al-Ghanayem and Joseph, 2020). Amongst these, there is only one produced by another *Psychrobacter* organism, *P. proteolyticus* DSM 13887^T^ (associated with Antarctic krill); similarly to ours, this enzyme showed highest activity in the temperature range 20–30°C, though at a more neutral pH 6.5-7.0 (Denner et al., 2001).

The genus *Psychrobacter*, having colonized a variety of cold habitats (terrestrial, marine and also host-associated) over a wide geographic distribution (Arctic, Antarctic, high altitude), is ideal to examine the effect of environmental adaptation on functional biodiversity while reducing the phylogenetic effect. Our comparative analysis of 22 homologous protease-coding genes from different *Psychrobacter* strains shows several clusters correlating with the habitat, for example the deep-sea marine cluster (*Psychrobacter* sp. choline-3u-12, *P. piscatorii* LQ58, *P. pacificensis* DSM 23406 and *Psychrobacter* sp. AntiMin-1, 97–98% sequence similarity), the marine organism-associated cluster (*Psychrobacter* sp. JCM 18902, *Psychrobacter* sp. JCM 18903 and *Psychrobacter* sp. P11F6) and the terrestrial-permafrost cluster (*P. arcticus* 273-4 and *P. cryohalolentis* K5). The protease of our strain *Psychrobacter* sp. 94-6PB was most similar to *P. urativorans* R310.10 and both organisms shared the same environmental source, being isolated from Antarctic soil. Thus, our data on the correlation between protease diversity and habitat further supports recent findings pointing toward the ecological specialization of *Psychrobacter* organisms (Zhang et al., 2017; Bakermans, 2018). For example, based on whole genome comparison, aquatic and terrestrial strains seem to be better adapted to low temperatures and high salinity than host-associated ones (Bakermans, 2018). It is conceivable that such adaptive traits are then expressed also in proteins and enzymes. While a clearer understanding can be gained as more genomes and comparative analyses become available, the aspect of functional microdiversity holds a great potential to refine the discovery of biomolecules of biotechnological interest.

Experimentally, we applied a recombinant DNA technique, in which we first identified the biosynthetic gene of interest via *in silico* comparative analysis of *Psychrobacter* genomes deposited in public databases, PCR-amplified the target gene in our strain 94-6PB and then cloned, expressed and purified it in heterologous *E. coli* host cells. Expression systems for heterologous production of cold-active enzymes have long suffered of various technical constraints (e.g., intrinsic low thermostability of the recombinant enzyme, protein misfolding), but new tools are becoming accessible to tackle these issues (Bjerga et al., 2016). In our specific case, the expression of the protease P94-6PB did not really benefit from the use of *E. coli* ArcticExpress (engineered to co-express Cpn60 and Cpn10 chaperons from *Oleispira antarctica*) compared to a standard *E. coli* BL21(DE3). Likewise, other enzymes from *Psychrobacter* isolates, including nitroreductase (Wang et al., 2019), lipase (Xuezheng et al., 2010), esterase (Novototskaya-Vlasova et al., 2012), have been successfully produced in heterologous *E. coli* cells.

Although not yet fully established, the genome mining/heterologous expression-based approach is becoming more and more popular, as it offers distinct advantages. First, it bypasses the cultivation step, which is often challenging for extremophilic microorganisms. Second, virtually any gene of interest available in public repositories can be simply synthesized and directly used for heterologous expression, with no need for the original organism. Third, having the enzyme-coding sequence at hand opens to further additional study possibilities; for example, it allows running sequence-based modeling to predict *in silico* enzyme activity and characteristics prior of testing, or conduct genetic engineering experiments to improve or customize the enzyme. Finally, it enables to investigate the enzyme microdiversity, which may arise within phylogenetically related groups of microorganisms as a consequence of the adaptation to different environmental niches (Zimmerman et al., 2013; Nguyen et al., 2019). Today, it is estimated that approximately 90% of enzymes used in industrial processes are recombinant forms (Adrio and Demain, 2014).

Thus, in this work we presented three main novelty aspects for the bioprospecting field, namely the use of extremophilic organisms, of genome repositories as a direct source of new functional biomolecules and the habitat-driven microdiversity to further expand the range of molecular variants. Further research in all these research areas is necessary to boost the utilization of microbial biotechnologies at industrial scale.

## Data Availability Statement

The data generated for this study can be found in the GenBank MN606318.

## Author Contributions

AP, DW, and NB conceived the study. E-LN, GF, and AP conducted experimental work and data analysis. AP and GF wrote the manuscript. All authors read and approved the manuscript and contribute to interpret the results.

## Conflict of Interest

The authors declare that the research was conducted in the absence of any commercial or financial relationships that could be construed as a potential conflict of interest.

## Footnotes

1 https://software.broadinstitute.org/morpheus
