# Supplementary material for: Discovery and Characterization of a New Cold-Active Protease From an Extremophilic Bacterium via Comparative Genome Analysis and in vitro Expression
Source: Front Microbiol. 2020 May 13;11:881. doi: 10.3389/fmicb.2020.00881 (PMC7247812; doi:10.3389/fmicb.2020.00881)

SUPPLEMENTARY MATERIAL - Discovery and characterization of a new cold-active protease from an extremophilic bacterium via comparative genome analysis and *in vitro* expression (Perfumo et al. 2020)

Table S1 Overview of bacterial strains used in this study, taxonomic identification and qualitative assessment of enzymatic activities (proteolytic and lipolytic, with further discrimination between esterases and lipases) in the temperature range of 4-18°C.

No.	Bacterial strains	16S rRNA sequence similarity %	Proteolytic activity	Lipolytic activity	Esterases	Lipases
**Phylum Actinobacteria**						
1	*Aeromicrobium ginsengisoli* 27-6PB	99		✓		✓
2	*Arthrobacter humicola* 07-06PB	99	✓	✓		✓
3	*Arthrobacter humicola* 27-02PB	96		✓		✓
4	*Arthrobacter ramosus* 17-07PB	99		✓		✓
5	*Arthrobacter stackebrandtii* 28-2PB	97	✓	✓	✓	✓
6	*Arthrobacter oryzae* 26-9PB	99	✓	✓	✓	
7	*Arthrobacter oxydans* 12-01PB	99	✓	✓	✓	
8	*Cryobacterium psychrotolerans* 15-01bPB	99				
9	*Demetria terragena* 13-07PB	94		✓		✓
10	*Demetria terragena* 14-07PB	93		✓		✓
11	*Frigoribacterium* sp. 25-14PB	98				
12	*Frigoribacterium faeni* 27-4PB	98				
13	*Humicoccus flavidus* 93-01PB	97		✓	✓	
14	*Leifsonia kafniensis* 27-3PB	98				
15	*Streptomyces psammoticus* 10-1PB	98		✓		
16	*Streptomyces herbaricolor* 08-02PB	98		✓	✓	✓
17	*Streptomyces clavifer* 11-03PB	98		✓		✓
**Phylum/class gamma-proteobacteria**						
18	*Pseudomonas frederiksbergensis* 18-02PB	99	✓	✓		
19	*Pseudomonas mandelii* 18-01PB	100	✓	✓	✓	✓
20	*Pseudomonas meridiana* 25-13PB	99	✓	✓	✓	✓
21	*Pseudomonas migulae* 20-02PB	99	✓	✓	✓	✓
22	*Pseudomonas thivervalensis* 18-03PB	99	✓	✓		✓
23	*Psychrobacter glacincola* 94-6PB	98	✓	✓		✓
24	*Rhodanobacter ginsengisoli* 28-4PB	99		✓		
**Phylum/class alpha-proteobacteria**						
25	*Brevundimonas subvibrioides* 94-07PB	99		✓		
26	*Methylobacterium organophilum* 05-05PB	97		✓		✓
27	*Sphingomonas aerolata* 25-8PB	99		✓		✓
28	*Sphingomonas meloni* 05-07PB	98	✓	✓		✓
29	*Sphingomonas oligophenolica* 27-1PB	98		✓		
**Phylum Bacteroidetes**						
30	*Chryseobacterium marinum* 15-04PB	98		✓		✓
31	*Pedobacter composti* 26-12PB	96				
32	*Salinibacterium xinjiangense* 28-7PB	98		✓	✓	✓
**Phylum Deinococcus**						
33	*Deinococcus claudionis* 28-1PB	99		✓		

Table S2 List of genomes of *Psychrobacter* strains harboring homologous genes coding for an extracellular protease that were used for comparative analysis in this study.

No.	*Psychrobacter* sp.	Accession	Region (bp)	Gene locus tag
1	*P. aquaticus* CMS56	AUSW01000030.1	149178 to 151391	M917_1730
2	*P. arcticus* 273-4*	NC_007204.1	342393 to 344603	PSYC_RS01465
3	*P. cryohalolentis* K5*	NC_007969.1	372014 to 374224	PCRYO_RS01610
4	*P. glacincola* BNF20*	LIQB01000001.1	60081 to 62288	AMK37_RS00245
5	*P. lutiphocae* DSM21542*	NZ_KB907628.1	80577 to 82763	H140_RS0104250
6	*P. pacificensis* DSM23406	FNAL01000001.1	178827 to 181004	SAMN05660405_00155
7	*P. piscatorii* LQ58	LNDJ01000053.1	35377 to 37554	AS194_06220
8	*P. urativorans* R10.10B*	NZ_CP012678.1	1862745 to 1864976	AOC03_RS08025
9	*Psychrobacter* sp. 4Dc	PJAT01000134.1	70914 to 73121	CXF61_12220
10	*Psychrobacter sp.* AC24	AYXM01000014.1	89917 to 92120	n.a.
11	*Psychrobacter* sp. C20.9	MRYC01000025.1	12337 to 14550	BTW00_12985
12	*Psychrobacter* sp. DAB_AL32B	NEXU01000052.1	64665 to 66877	CAN34_06130
13	*Psychrobacter* sp. ENNN9_III	LNUO01000025.1	35539 to 37749	n.a.
14	*Psychrobacter* sp. G*	NC_021661.1	371405 to 373615	PSYCG_RS01570
15	*Psychrobacter* sp. JCM 18902*	BAWI01000001.1	459222 to 461434	JCM18902_419
16	*Psychrobacter* sp. JCM 18903*	BAWJ01000020.1	40552 to 42759	JCM18903_2750
17	*Psychrobacter* sp. P11F6*	LJCE01000001.1	441754 to 443961	AK822_RS01875
18	*Psychrobacter* sp. P11G3*	LJCF01000001.1	418193 to 420400	AK824_RS01750
19	*Psychrobacter* sp. PAMC21119*	NZ_AHVZ01000041.1	142936 to 145146	RH96_RS14040
20	*Psychrobacter* sp. PRwf-1*	NC_009524.1	376938 to 379145	PSYCPRWF_RS01590

Gene sequences in strains marked with (*) were used to design PCR/sequencing primers for this study.
n.a. not available.

**Table S3 List of primers used to PCR-amplify the protease-coding gene in *Psychrobacter* sp. 94-6PB.**				
**Primer name**	**Primer sequence (5' to 3')**	**$T_{(a)}$ (°C)**	**Forward (F) or reverse (R)**	**Binding position (bp)**
P1	CGYAGCGTGTAAGATTG	46 °C	F	5' UTR (300 upstr)
P4	GTRACVCCTGAYTCTTCTTG	46 °C	R	1133
P5	CAAGAAGARTCAGGBGTYAC	46 °C	F	1112
P6	TTDATBGTATCCCAAGGCA	46 °C	R	1780
P9	CTGGATAACCTAGCATTGG	46 °C	F	1618
P10	GTTTGATTCGTGGTAAGC	46 °C	F/R	1027 (F); 2183 (R)
P20	AGCCTTATCATCCTTAATCAGC	49 °C	R	3' UTR (203 downstr)
P24	ACAGCAAGATCCGCAGTTCG	49 °C	F	1862
P26	GCTTAACTAGTATCAACACTGCTG	49 °C	F	5' UTR (54 upstr)

Figure S1 Plate screening assays for the detection of proteolytic and lipolytic activities in isolates of Antarctic bacteria. Calcium caseinate agar plate with spotted bacterial cultures tested for the synthesis of extracellular proteases at 18ºC (strains R2, Co1 and M1 positive, strains O2 and X1 negative) (A). Triolein-rhodamine B agar plate with a spotted bacterial culture (strain F3) positive for the synthesis of extracellular lipase at 18ºC (B).

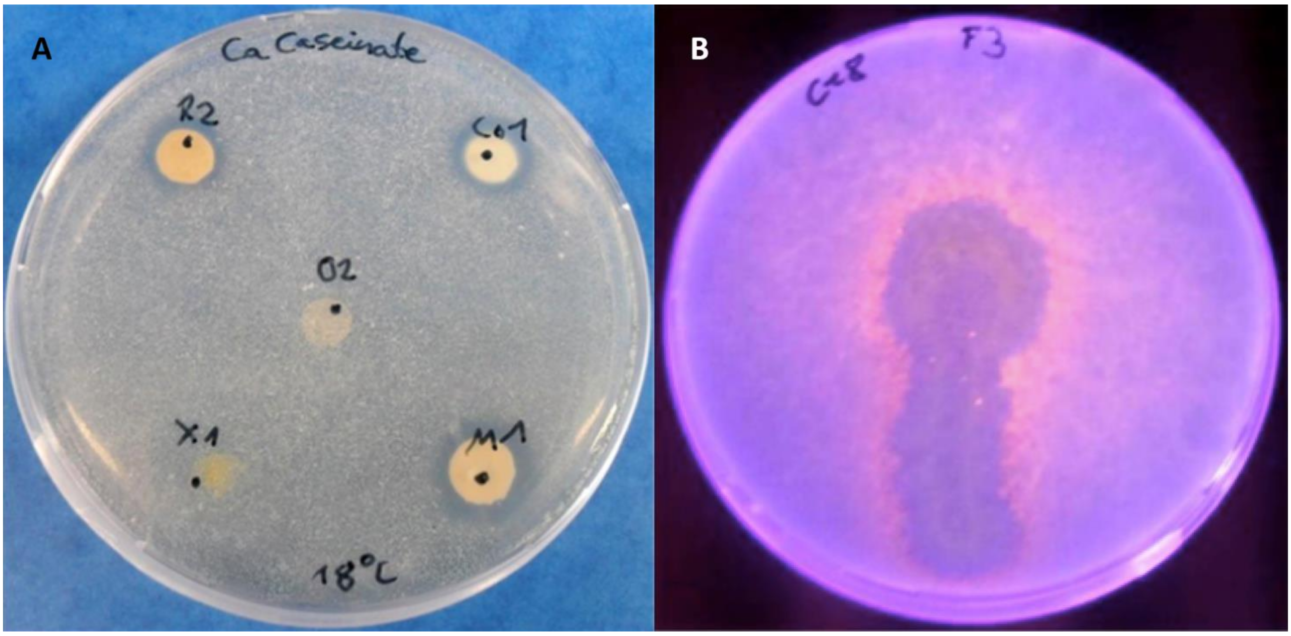

Figure S2 Images of agarose gels showing the protease gene of *Psychrobacter* sp. 94-6PB amplified in three main overlapping fragments and as full length amplicon. Fragment 1 was PCR amplified with primers P1-f + P6-r (product size 2080 bp); fragment 2 with primers P10-f + P10-r; product size 1156 bp; fragment 3 with primers P24-f + P20-r (product size 555 bp); the full length gene including upstream and downstream regions was obtained with primers P26-f + P20-r (product size 2471 bp). Molecular marker is shown on the left hand side of the gels.

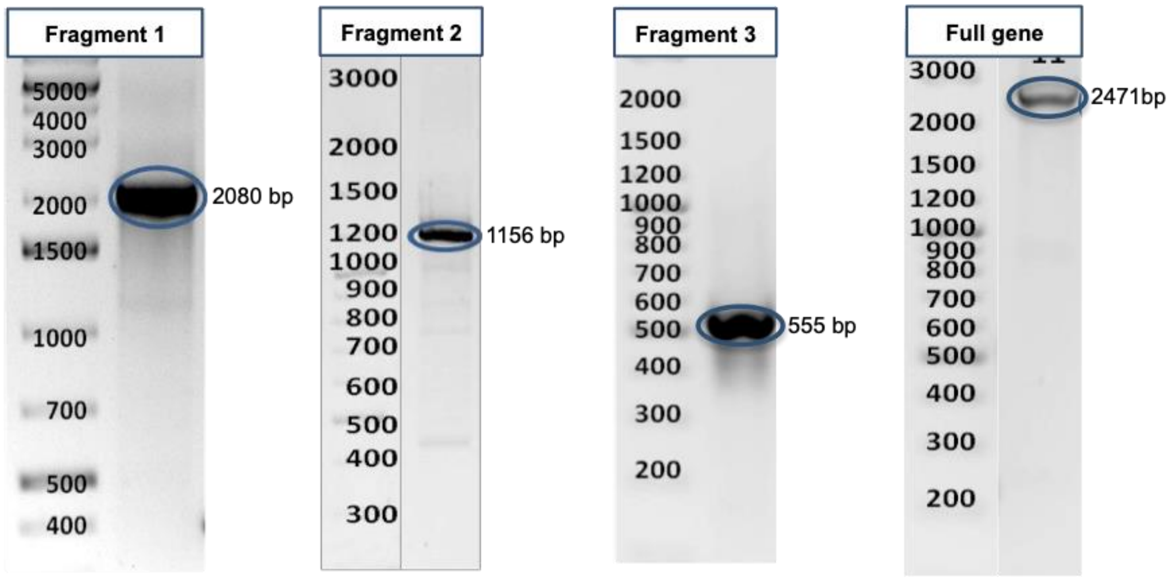

Figure S3 Molecular phylogenetic analysis of serine protease enzymes in *Psychrobacter* bacteria. The evolutionary history was inferred by using the Maximum Likelihood method based on the Tamura-Nei model. The tree with the highest log likelihood (-21424.39) is shown. The percentage of trees in which the associated taxa clustered together is shown next to the branches. Initial tree(s) for the heuristic search were obtained automatically by applying Neighbor-Join and BioNJ algorithms to a matrix of pairwise distances estimated using the Maximum Composite Likelihood (MCL) approach, and then selecting the topology with superior log likelihood value. The tree is drawn to scale, with branch lengths measured in the number of substitutions per site. The analysis involved 22 nucleotide sequences of *Psychrobacter* spp. from terrestrial (brown colored), aquatic (blue colored) and host-associated (red colored), and *Moraxella lincolnii* CCUG 9405 as outgroup. Codon positions included were 1st+2nd+3rd+Noncoding. All positions containing gaps and missing data were eliminated. There were a total of 1947 positions in the final dataset. Evolutionary analyses were conducted in MEGA7.

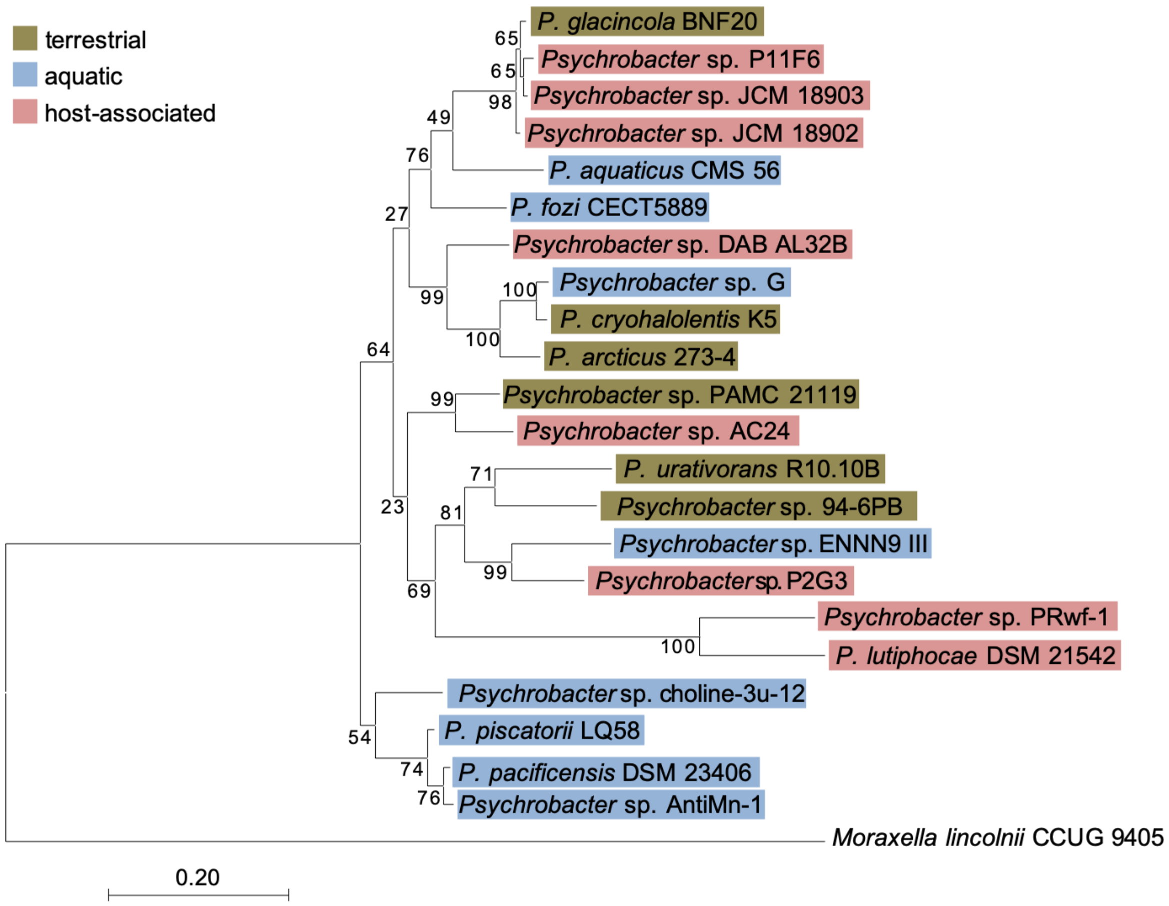

Supplement: Supplementary file 1 [file Data_Sheet_1.pdf]
